# Drug discovery from Nature: automated high-quality sample preparation

**DOI:** 10.1155/S1463924600000249

**Published:** 2000

**Authors:** Ralf Thiericke

**Affiliations:** Hans-Knöll-Institute for Natural Products Research Beutenbergstraße 11 Jena D- 07745 Germany

## Abstract

Secondary metabolites from plants, animals and microorganisms have been proven to be an outstanding source for new and innovative drugs and show a striking structural diversity that supplements chemically synthesized compounds or libraries in drug discovery programs. Unfortunately, extracts from natural sources are usually complex mixtures of compounds:: often generated in time consuming and for the most part manual processes. As quality and quantity of the provided samples play a pivotal role in the success of high-throughput screening programs this poses serious problems. In order to make samples of natural origin competitive with synthetic compound libraries, we devised a novel, automated sample preparation procedure based on solid-phase extraction (SPE). By making use of a modified Zymark RapidTrace^®^ SPE workstation an easy-to-handle and effective fractionation method has been developed which allows the generation of highquality samples from natural origin, fulfilling the requirements of an integration into high-throughput screening programs.


Journal of Automated Methods & Management in Chemistry, Vol. 22, No. 5 (September–October 2000) pp. 149–157

### Drug discovery from Nature: automated high-quality sample preparation

**Ralf Thiericke**

*Hans-Knöll-Institute for Natural Products Research, Beutenbergstraße 11, D-07745, Jena, Germany. e-mail: thierick@pmail.hki-jena.de*

*Secondary metabolites from plants, animals and microorganisms have been proven to be an outstanding source for new and innovative drugs and show a striking structural diversity that supplements chemically synthesized compounds or libraries in drug discovery programs. Unfortunately, extracts from natural sources are usually complex mixtures of compounds: often generated in time consuming and for the most part manual processes. As quality and quantity of the provided samples play a pivotal role in the success of high-throughput screening programs this poses serious problems. In order to make samples of natural origin competitive with synthetic compound libraries, we devised a novel, automated sample preparation procedure based on solid-phase extraction (SPE). By making use of a modified Zymark RapidTrace® SPE workstation an easy-to-handle and effective fractionation method has been developed which allows the generation of high-quality samples from natural origin, fulfilling the requirements of an integration into high-throughput screening programs.*

### Introduction

Concepts to improve the generation and identification of lead structures are key issues in the development of new pharmaceuticals and agro-chemicals. In the early stages of drug discovery high sample numbers are requested to feed the current capacities of the high-throughput screening (HTS) machinery. At present, compound libraries from combinatorial chemistry are the major source for HTS programs. On the other hand, however, nature has been proven to be an outstanding source for new and innovative drugs [1]. Numerous examples from medicine, such as cyclosporin A, lovastatin, acarbose, paclitaxel, FK506, topotecan, or miglitol, impressively demonstrate the innovative potential of natural products regarding their impact on progress in drug discovery and their importance on the drug market [1]. At present, natural products and substances derived from the account for about 30% of the worldwide sales of drugs and 9 of the top 20 best selling drugs.

Secondary metabolites from plants, animals and microorganisms show a striking structural diversity and supplement chemically synthesized compound collections or libraries in drug discovery programs. We presume that the molecular understanding of complex biological communication networks will have a dramatically influence on the discovery processes of new drugs (figure 1) [2]. Hypothetically, diseases correlated to complex molecular interactions afford structurally more complex drug candidates which points to the evolutionary proven, stereochemically defined, and, in comparison with combinatorials, often structurally more complex natural products.

### Access to structural diversity from Nature

For a number of reasons natural products passed through a phase of reduced interest in drug discovery strategies (figure 2). As only a few thousand pure natural compounds are available for screening, there is a need for new and more efficient strategies to access structural diversity from Nature. At present, strategies to improve the techniques for isolation and structural characterization of natural products are far from obtaining hundreds of thousands of well characterized pure compounds [3], because the identification, purification and structural characterization of individual compounds from complex mixtures are cost- and time-consuming procedures. In order to take advantage of biodiversity in combinatorial chemistry, approaches to enhance natural products or to direct a total synthesis of a natural product towards combinatorial structure variation are currently under way [4]. However, the latter strategies depend on the availability of either pure or biologically striking natural products. Therefore, at present, extracts from natural sources play the major role in drug discovery from Nature, but such samples have a number of difficulties (see figure 2) as follows.

- Access to biological material from plants, microorganisms, fungi, animals, etc. has to be quaranteed. However, the amount of secondary metabolites wanted may vary from sample to sample.
- Extracts from natural sources are often generated in time-consuming and, for the most part, manual processes.
- The extracts usually contain a complex mixture of substances. These mixtures can result in overlapping biological effects, especially in functional assay systems. Thus, biologically active compounds may be hidden or false-positive effects may result from a combination of different compounds, not allowing reliable 'go/no–go' decisions for further processing.
- Recognition and de-replication of known, already isolated, or unwanted types of compound are often not possible until a late stage of the investigative process.
- Consistency and viscosity of the extracts are not well suited for the requirements of automated liquid handling systems.

Consequently, there exists a need for improving the technologies of sample preparation from natural sources in order to produce high-quality test samples with less complexity and more reliable reproducibility. In

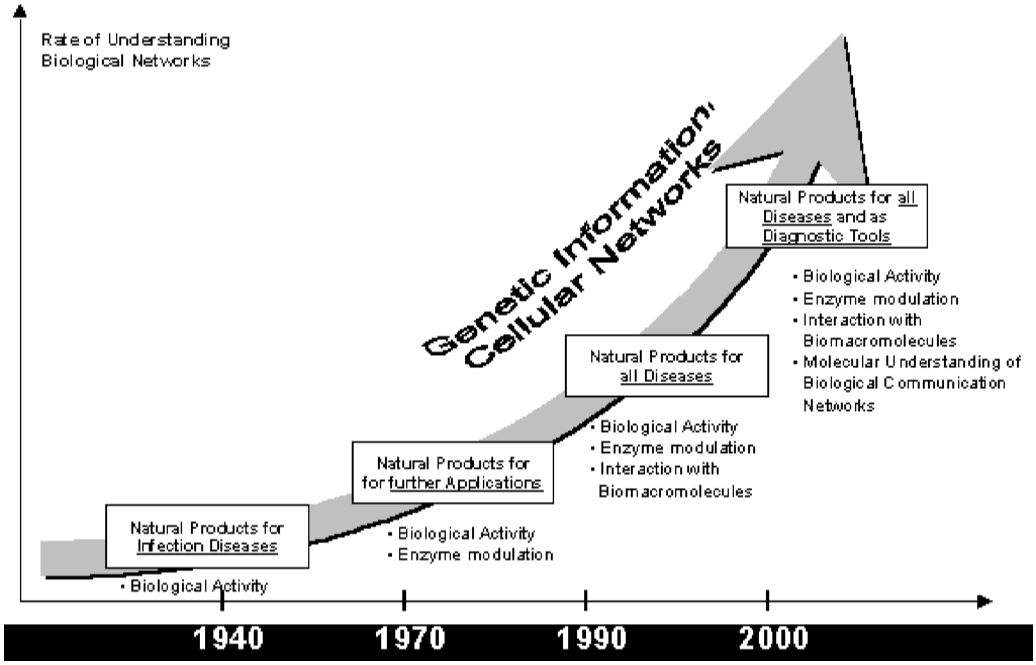

*Figure 1. Natural products: from biological activity to a molecular understanding of biological communication networks* [2].

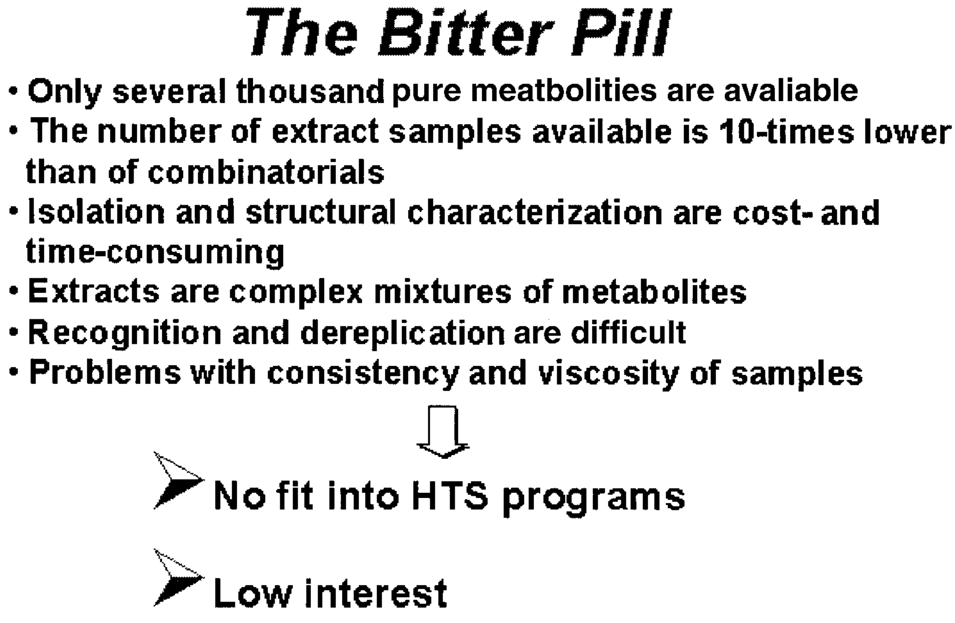

*Figure 2. Challenges for drug discovery approaches with samples from natural sources.*

addition, the tedious, time-consuming and manual processes of sample preparation have to be replaced to provide samples of sufficient quality and quantity for high-throughput screening programs.

### The single-step solid-phase extraction approach

Our approach [5] evolved from a procedure for sample preparation from microbial broths which had already been developed for chemical screening [6]. This manual one-step procedure works by adsorption of the secondary metabolites present in the culture broth of microbial cultivations on to polystyrene resins such as Amberlite XAD-16 and elution with organic solvents to yield concentrated complex mixtures of natural products [7, 9]. In order to achieve a significantly higher quality of the samples, chromatographic fractionation on new adsorption resins with enhanced chromatographic features as well as higher adsorption capacities is of fundamental importance. The results below have been achieved with modified Zymark RapidTrace® SPE workstations.

Most critical for a high-resolution fractionation of complex material from natural sources is the choice of the solid-phase extraction (SPE) material. Making use of

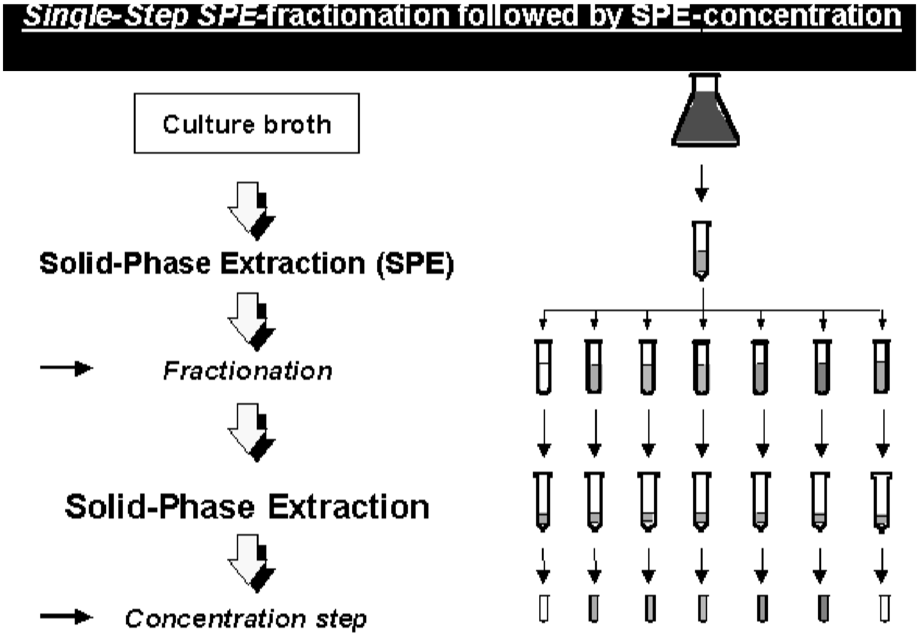

*Figure 3. Single-step SPE method used for the evaluation of the SPE-adsorption resins.*

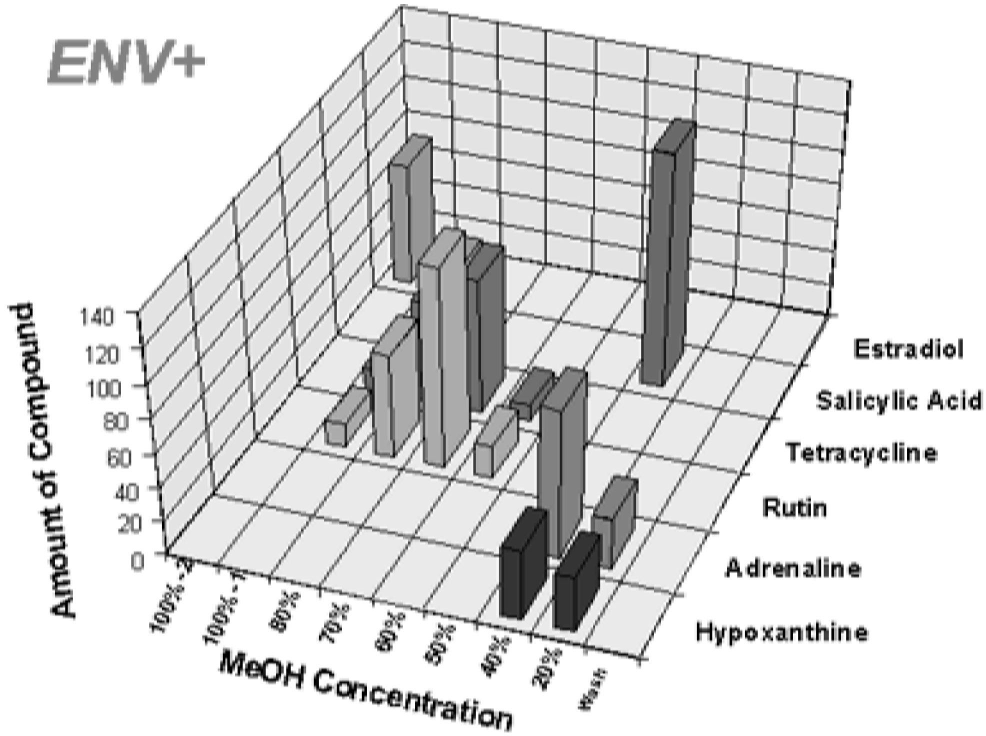

*Figure 4. Separation of the mixture of natural products by single-step SPE fractionation and concentration with the adsorption resin ENV$^+$ (International Sorbent Technology Ltd, Mid Glamorgan, UK).*

both a single-step fractionation method that is followed by a concentration step also based on SPE (figure 3), and a mixture of pure natural products with a broad range of polarity a number of adsorption resins and reverse-phase silica gel materials were evaluated. Among the resins tested [5], ENV$^+$ showed the broadest spectrum of adsorption, in combination with a well resolved chromatographic separation of the compounds in the first fractionation step (figure 4) [9].

On the basis of the ENV$^+$ adsorption resin a single-step fractionation and concentration method was developed and optimized which allows fractionation of the secondary metabolites present in 50 ml of culture broth from *Actinomycetes* and *Fungi imperfecti* (see figure 5) [9]. After cultivation of the microorganisms the culture broth was centrifuged and the supernatant was applied to ENV$^+$ (500 mg). Then the resin was washed with water and the secondary metabolites were eluted with increasing

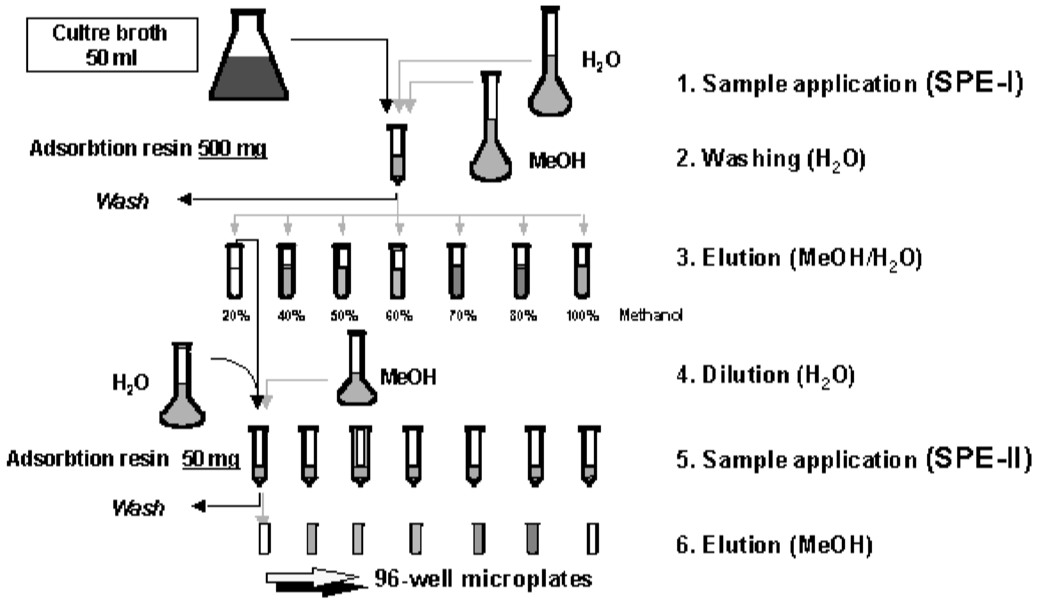

*Figure 5. Optimized method for single-step SPE fractionation and concentration of material from natural sources. SPE-I: chromatographic fractionation with SPE resins. SPE-II: Sample concentration* via *SPE resins.*

amounts of methanol (20%, 40%, 60%, 70%, 80%, and twice 100%). The obtained fractions were diluted with water to yield methanol concentrations of about 2–5% which were pumped on to a second SPE column (50 mg of resin) for concentration. The best results concerning the concentration of compounds were achieved if the choice of SPE materials was adjusted to the elution characteristic in the preceding fractionation. For those fractions eluted from the ENV$^+$ resin with 50% or less methanol, ENV$^+$ was most efficient also for the sample concentration procedure. The more lipophilic fractions, eluted with more than 50% methanol, were best adsorbed and eluted from RP-C$_8$ material. After a washing step with water, the adsorbed organic compounds were eluted with *ca.* 500 µl of organic solvent into sample vials that fitted into the 96-well plate format. In the last elution step either methanol and other alcohols or even DMSO (dimethylsulphoxide) can be used. Because of the physico-chemical behaviour of each substance, the concentration factors vary from 4-times to 40-times [9]. With slight variations in the handling parameters this procedure can also be adapted to the fractionation of other organisms such as microalgae [10].

To acquire information about the sample quality from the single-step SPE fractionation and concentration method compared with the manual one-step procedure without fractionation on Amberlite XAD-16 resin, a number of culture broths from various microbial cultivations were analysed. In figure 6 the MALDI-TOF mass spectrometry analysis of samples obtained from *Streptomyces* sp. strain GT 61174 are depicted. The sample obtained from the XAD-16 procedure showed *ca.* 15 to 20 different metabolites while single-step fractionation and concentration into seven extracts enriched the compound, with $m/z = 474.8$.

In addition, samples from single-step SPE fractionation exhibit advantages in biological screening, especially for the generation of efficient 'hit-lists'. On the basis of a functional yeast-based transcription assay with the human progesterone receptor [11], a number of samples from the XAD-16 extraction method were screened for biological activity and samples from the *Streptomyces* strains GT 51237/2, GT 41186/2, and GT 41179/3 showed activity. In parallel, samples from the cultivation of these strains obtained by single-step SPE fractionation and concentration (resin: ENV$^+$) were prepared and analysed. As depicted in figure 7, the recovery rates of the biological activity in GT 51237/2 and in GT 41186/2 are nearly identical to those from the XAD-16 method, while in the case of GT 41179/3 parts of the biologically active principle remained on the SPE column. In our studies some highly lipophilic compounds were found to stick to the resin even when pure organic eluent was used—a disadvantage of the ENV$^+$ resin. In addition, GT 41186/2 produces two different biologically active metabolites (elution with 40% and 100% methanol) which, in principle, are not detectable in XAD-16 samples. A further advantage lies in the possibility of the separation of wanted biological activity (here: interaction with the human progesterone receptor) from, for example, toxic compounds, which further increases the quality towards that of the hit-lists.

### The multi-step single-phase extraction approach

In order to improve the separation quality towards pure compounds, the SPE protocol was extended by a second fractionation step. The focus lies on those cases where more complex samples have to be handled or further fractionation is desirable for identification of biologically active compounds. The developed procedure can be outlined as follows (figure 8). In a first step, the aqueous crude compound mixture is applied on to a first SPE cartridge. Fractions of the column are eluted by stepwise

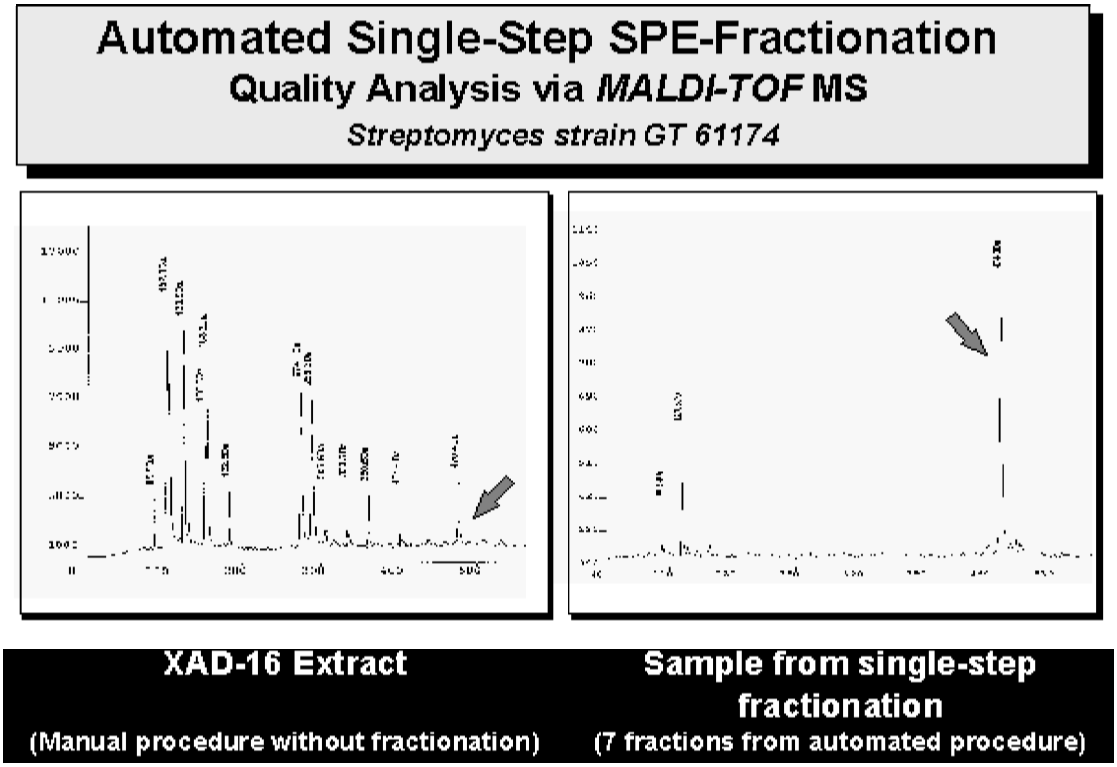

*Figure 6. Quality analysis of samples obtained from the cultivation of* Streptomyces sp. (*strain GT 61174*) via *MALDI-TOF mass spectrometry.*

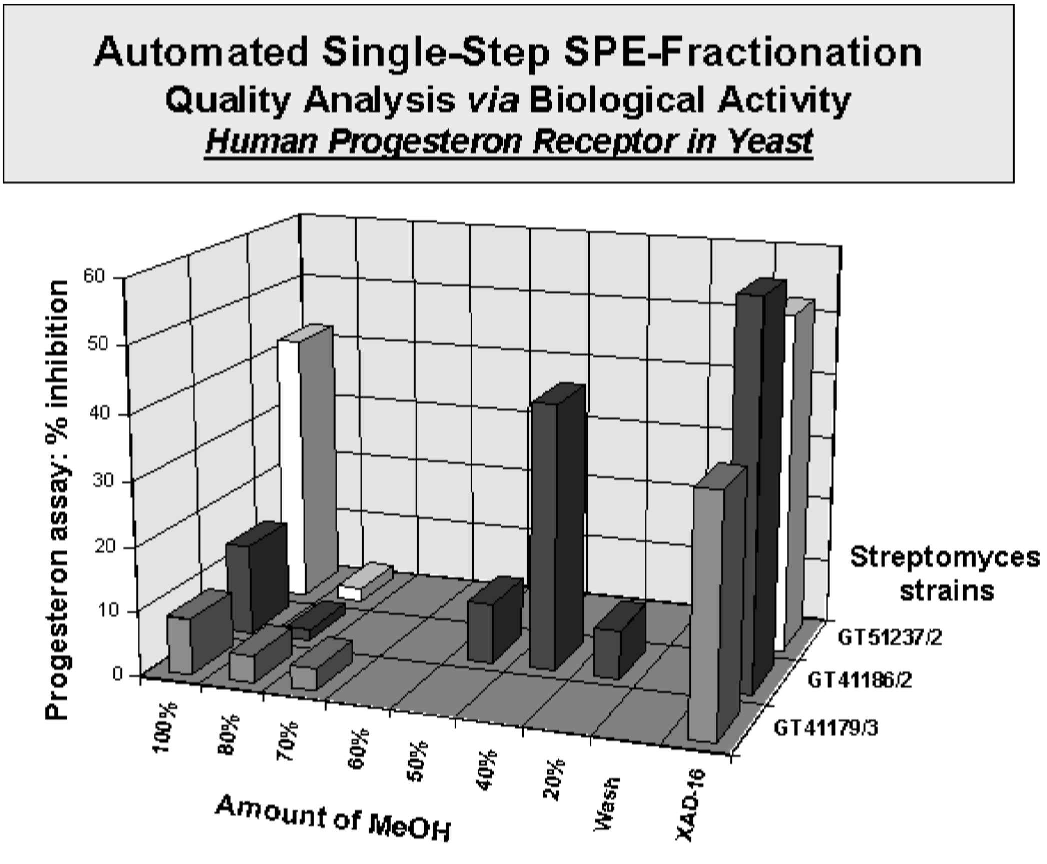

*Figure 7. Quality analysis of samples prepared from the cultivation of* Streptomyces *strains* via *biological screening using a yeast-based transcription assay with the human progesterone receptor* [11].

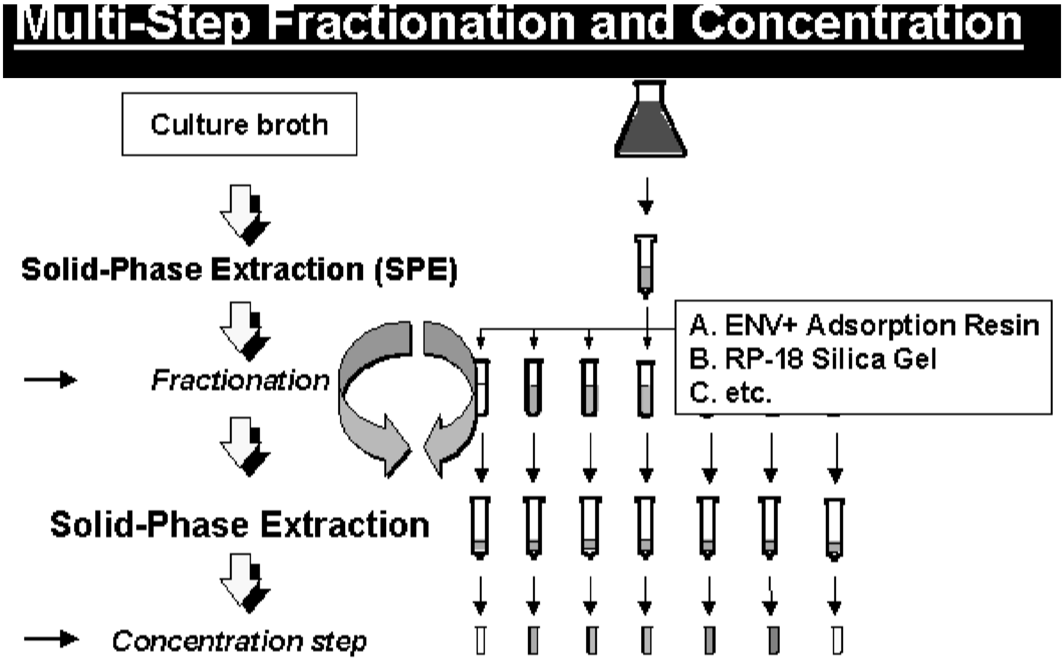

*Figure 8. Multi-step SPE for efficient fractionation and concentration of material from natural sources making use of different adsorption resins.*

application of water/organic solvent or buffer/organic solvent mixtures with an increasing content of organic solvent. The fractions generated in this first step can be further fractionated by using an identical procedure. However, before re-loading on to the next column, the solution has to be diluted with water in order to decrease its elution strength. As for the concentration step in the single-step fractionation procedure the chromatographic material has to be chosen according to the type of fraction yielded from the first separation. After the final fractionation, the samples need to be concentrated for further processing, for example for integration into HTS.

As an example, the culture filtrate of the fungal strain GT 076111 was fractionated according to the multi-step SPE fractionation protocol. HPLC spectra (diode array detector at a wavelength of 254 nm) of representative fractions illustrate the suitability of the method to prepare samples of significantly reduced complexity (figure 9). A spectrum of an extract prepared by a single-step work-up procedure with Amberlite XAD-16 (figure 9 (A)) is compared with spectra of fractionated samples from the same strain prepared by automated multi-step SPE (figures 9 (B) and (C)). The multi-step SPE protocol included two fractionations and one concentration step. Figure 9 (B) shows the spectrum of one of the fractions after the first fractionation step. The eluent was further fractionated and subsequently concentrated. In 9 (C) the sample from 9 (B) has been submitted to an additional fractionation step.

### Automation with Zymark RapidTrace® SPE workstations

Automation of both the single-step and the multi-step SPE protocols require the following features:

(i) handling of liquid volumes larger (e.g. 50 ml) than SPE machines usually allow,
(ii) fraction collection is necessary,
(iii) re-loading of fractions from the collection device should be given,
(iv) the elution strength of fractions has to be reduced by dilution with water or buffer,
(v) elution volumes range from 5 ml to 300 µl, and
(vi) parallel handling should be possible.

A commercially available automation concept meeting all these requirements was not available. Closest was the Zymark RapidTrace® SPE workstation, an apparatus for automated SPE that has already been used as a stand-alone machine for method development (see above). Therefore, in close cooperation with Zymark GmbH (Germany) a workstation consisting of eight RapidTrace modules was re-designed according to our special requirements.

The modifications were set up as depicted in figures 10 and 11. One module is used to perform the first fractionation step. Specially designed tube connectors mounted into the standard tube rack provide samples for the other seven modules. The eluted fractions of the first fractionation are collected in special PTFE vessels, equipped with a magnetic stirrer. These mixing chambers with a capacity of 200 ml replace the standard 6 ml chambers and are used for dilution and solvent mixing, but also serve as fraction collectors. With a pneumatically driven vacuum generator these chambers can be set under low vacuum to ensure complete emptying of the supply tubes. After the first fractionation step, further processing (fractionation or concentration) of the samples is carried out in parallel on up to nine RapidTrace modules (at present, the number is limited by the control software). After the concentration step special rack adapters allow the

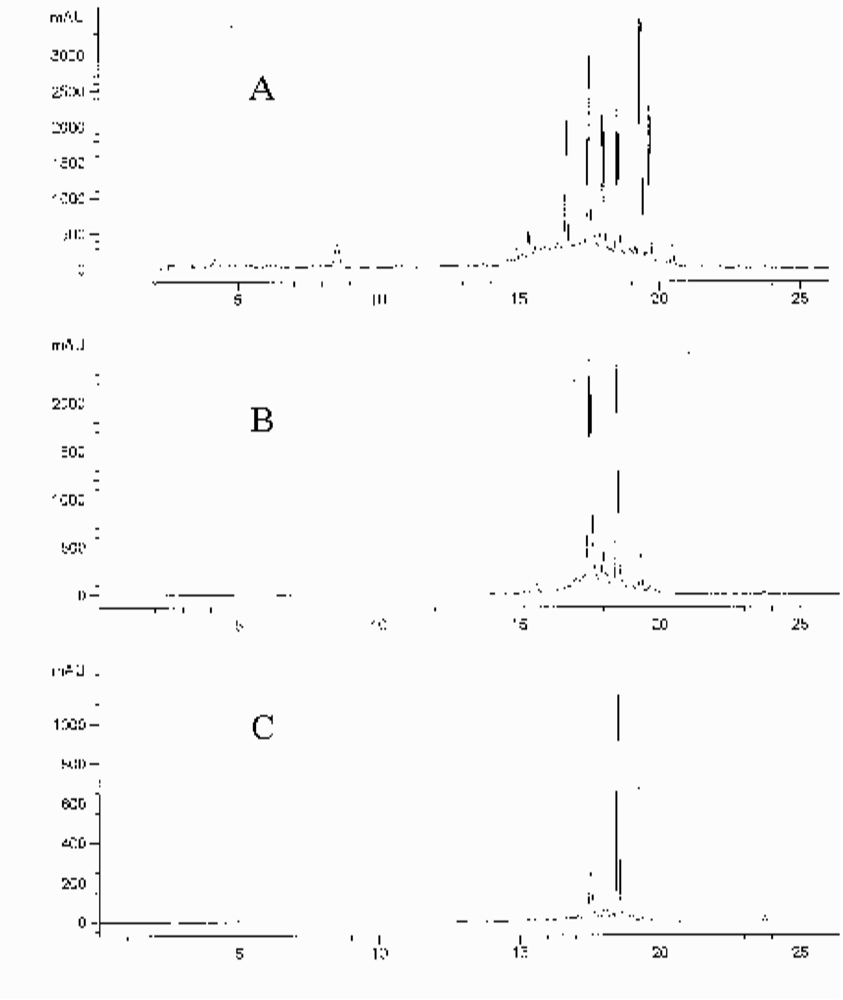

*Figure 9. HPLC-DAD spectra (at 254nm) of samples obtained from the cultivation of the fungal strain GT 076111: (A) Amberlite XAD-16 sample; (B) single-step fractionation; (C) multi (2)-step fractionation of sample (B).*

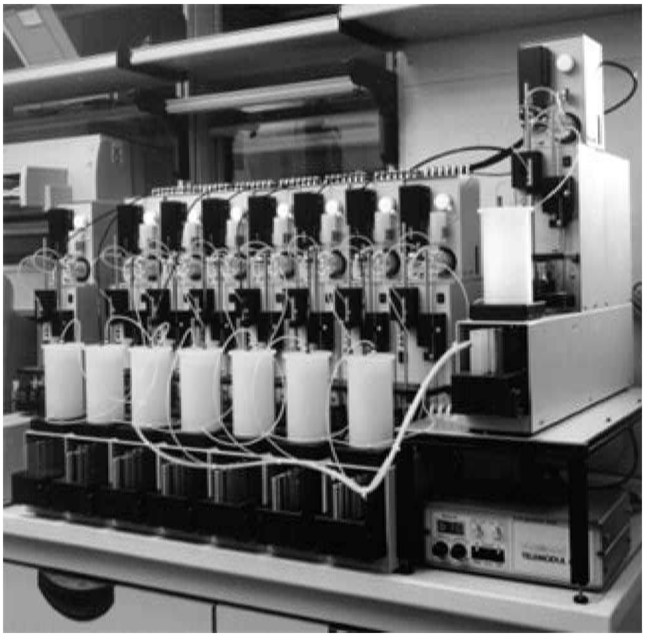

*Figure 10. The modified Zymark RapidTrace® SPE work-station developed for single-step and multi-step SPE fractionation and concentration.*

elution into microtubes that can be applied to deep well microplates.

### Conclusion

Samples from natural sources prepared by the automated single- and multi-step SPE fractionation methods were successfully introduced into both our biological and our physico-chemical screening programs [6]. In these screening approaches reduced complexity of the applied samples results in a strong gain in information. The advantage of fractionated samples is particularly obvious in biological screening. Assay results become more re-liable and reproducible if not a complex mixture but fractions of reduced complexity are submitted to HTS. In addition, fractionation might separate toxic components from interesting, biologically active ones [5, 9]. Figure 12 summarizes information about the quantity of samples which can be prepared from microbial broths with one modified RapidTrace® SPE workstation per year.

In our automation protocol the samples can be generated without (in case of one fractionation and concentration step) or with only minor manual intervention (changing

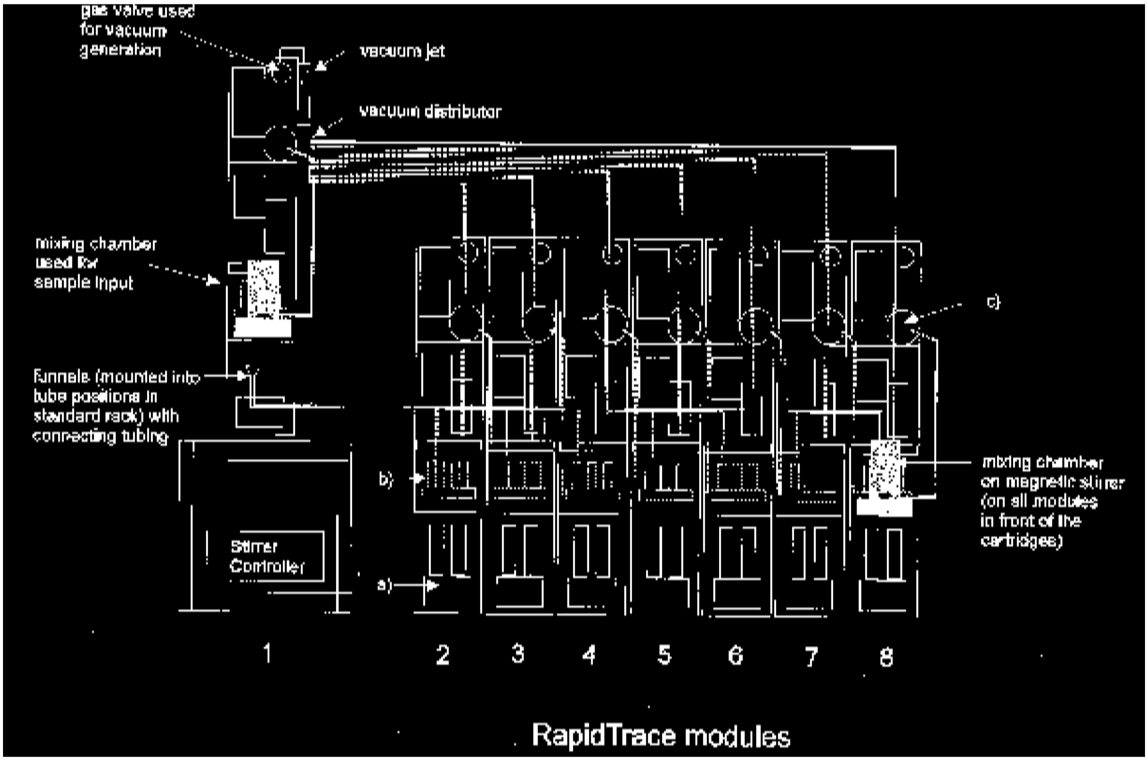

*Figure 11. Schematic drawing of the modified Zymark RapidTrace® SPE workstation. (a) Standard tube rack for 10 sample tubes and 10 collection tubes; (b) SPE column turret (holds up to ten 1 ml or 3 ml SPE cartridges); (c) 12-port valve for the distribution of the liquid flow.*

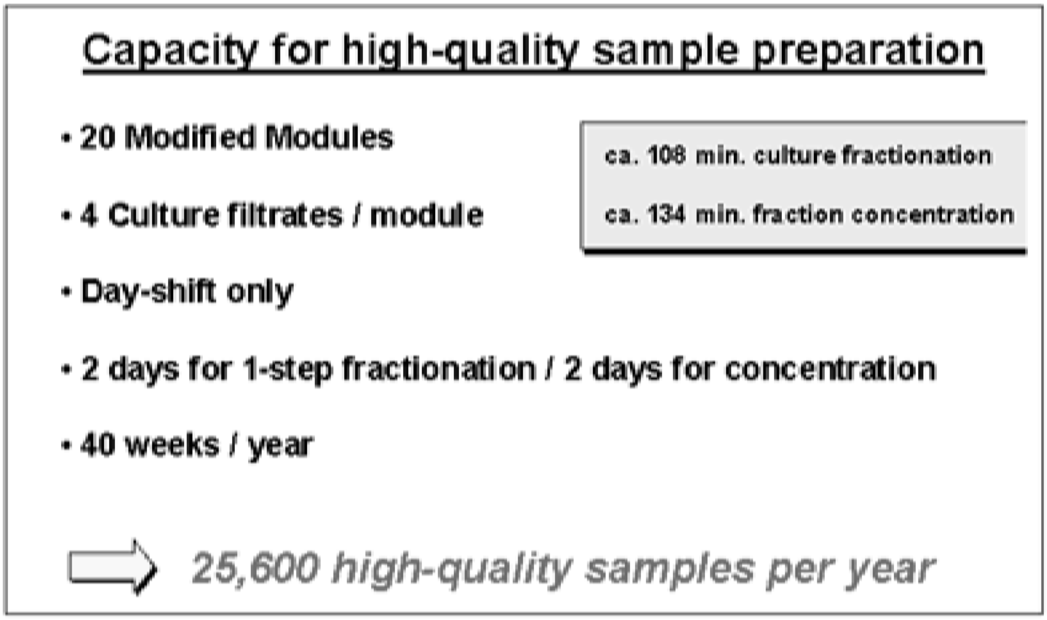

*Figure 12. Calculation of the capacity for high-quality sample preparation using the single-step SPE fractionation and concentration method with one modified RapidTrace® SPE workstation.*

tube positions in the sample rack if two or more fractionation steps are carried out). The set-up allows parallel processing of samples after the first fractionation step. However, present commercially available devices for parallel SPE in the 96-format, which is about to be a routine technique in diagnostic sample preparation [11], cannot be used for our method owing to the limited possibilities of handling liquid volumes of more than 2 ml per sample. In order to make high-quality samples from natural origin available in significantly higher numbers we are currently working on a concept to increase the grade of parallel processing adjusted to our needs.

### Acknowledgement

During her Ph.D. thesis, Dr I. Schmid developed the SPE methods for high-quality sample preparation from natural sources presented in this article. We thank Dr K. Geschwill and K. Kristant from Zymark GmbH, Germany, for valuable discussions and the construction of the modified Zymark RapidTrace® SPE workstation.

### References

1. Grabley, S. and Thiericke, R. (eds), 1999, *Drug Discovery from Nature*, (Berlin: Springer Verlag).
2. Hinnen, A., 1999, personal communication, (Hans-Knöll-Institute for Natural Products Research).
3. Waterman, P. G., 1998, in: *Advances in Drug Discovery Techniques* (Chichester: Wiley), pp. 13–23.
4. Paululat, T., Tang, Y.-Q., Grabley, S. and Thiericke, R., 1999, *Chemistry Today*, **17**, 52–56.
5. Schmid, I., Sattler, I., Grabley, S. and Thiericke, R., 1999, *J. Biomolec. Screening*, **4**, 15–25.
6. Grabley, S. and Thiericke, R., 1999, in: S. Grabley and R. Thiericke (eds) *Drug Discovery from Nature* (Berlin: Springer Verlag), pp. 124–148.
7. Bach, G., Breiding-Mack, S., Grabley, S., Hammann, P., Hutter, K., Thiericke, R., Wink, J. and Zeeck, A., 1993, *Liebigs Ann. Chem.*, 241–250.
8. Burkhardt, K., Fielder, H.-P., Grabley, S., Thiericke, R. and Zeeck, A., 1996, *J. Antibiotics*, **49**, 432–437.
9. Schmid, I., 1999, Ph.D. thesis, Technical University of Berlin.
10. Ebert, G., 1999, Ph.D. thesis, Technical University of Berlin.
11. Rudakoff, B., Undisz, K., Mayer, G., Sobek, L., Kaufmann, G., Thiericke, R., Grabley, S. and Munder, T., 1999, *J. Cell. Biochem.*, **73**, 126–136.
12. Kaye, B., Herron, W. J., Macrae, P. V., Robinson, S., Stopher, D. A. and Venn, R. F., 1996, *W. Wild. Anal. Chem.*, **68**, 1658–1660.

